# *SEPT14* Mutations and Teratozoospermia: Genetic Effects on Sperm Head Morphology and DNA Integrity

**DOI:** 10.3390/jcm8091297

**Published:** 2019-08-23

**Authors:** Ya-Yun Wang, Tsung-Hsuan Lai, Mei-Feng Chen, Hui-Ling Lee, Pao-Lin Kuo, Ying-Hung Lin

**Affiliations:** 1 Department of Chemistry, Fu Jen Catholic University, New Taipei City 24205, Taiwan; 2 Graduate Institute of Biomedical and Pharmaceutical Science, Fu Jen Catholic University, New Taipei City 24205, Taiwan; 3 Department of Obstetrics and Gynecology, Cathay General Hospital, Taipei 10630, Taiwan; 4 School of Medicine, Fu Jen Catholic University, New Taipei City 24205, Taiwan; 5 Bone and Joint Research Center, Chang Gung Memorial Hospital, Taoyuan County 83301, Taiwan; 6 Department of Obstetrics & Gynecology, National Cheng Kung University, Tainan 70101, Taiwan; 7 Department of Obstetrics and Gynecology, National Cheng-Kung University Hospital, Tainan 70403, Taiwan

**Keywords:** male infertility, teratozoospermia, septin, *SEPT14*, DNA damage

## Abstract

The main objective of this study was to evaluate the potential genetic effects of *SEPT14* on male infertility through sequencing the *SEPT14* coding region. To address this research gap, 254 men with sperm abnormalities and 116 normozoospermic men were recruited, and the whole-coding regions of *SEPT14* were sequenced. Two heterozygous mutations, p.Ala123Thr (3/254 vs. 0/116) and p.Ile333Thr (3/254 vs. 0/116), were identified in these cases. A high percentage of defective sperm heads was found in sperm with mutated *SEPT14*. Both mutations are highly evolutionarily conserved among vertebrates. The results of a fine morphological and chromatin structural analysis indicated severely malformed sperm heads with abnormal chromatin packaging through transmission electron microscopy and Toluidine blue staining. Compared with controls, high DNA fragmentation was demonstrated in sperm from cases carrying *SEPT14* mutations using the comet assay. In addition, these two mutations in *SEPT14* affected its polymerization ability in vitro. These data revels that the two *SEPT14* missense mutations impaired sperm head morphology and induced DNA damage. Our study suggests that genetic variant of *SEPT14* is one of the effects for human sperm formation and male fertility.

## 1. Introduction

### 1.1. Male Infertility and Sperm DNA Damage

Approximately 9% of couples are affected by infertility worldwide and male infertility accounts for 30% of couple infertility cases [1,2,3]. And, one of the major causes of male infertility is teratozoospermia, which is frequently accompanied with sperm DNA defects [4]. As well, sperm with high levels of DNA damage or abnormal DNA packaging has negative effects on embryo development and pregnancy outcomes, including pregnancy loss, recurrent spontaneous abortion, and lower live birth rates [5,6,7,8,9,10,11]. According to the various levels of sperm nuclear abnormalities, sperm DNA damage can be categorized as follows: (a) Damage to the actual DNA; (b) nuclear-related gene mutation or protein loss causes decreased DNA compaction; and (c) irregular chromatin structure [12,13,14,15]. To date, only mutations in certain genes have been linked to sperm DNA damage in a clinical aspect (i.e., *PROTAMINE*, *AURKC*, *SPATA16*, *PICK1*, *DPY19L2*, and *SEPT12*) [16,17,18,19].

### 1.2. Septins

Septins (SEPTs) are highly evolutionarily conserved GTP-binding proteins and are defined as the fourth component of the cytoskeleton [20,21]. All SEPTs polymerize as hetero-oligomeric structures, such as filaments and rings, and are involved in the regulation of cellular processes, including cytokinesis, cell polarity, and membrane dynamics, through cooperation with diverse cytoskeletons [20,21,22,23,24]. The dysfunction of human SEPTs is associated with the pathology of conditions such as cancer, neurological diseases, and male infertility [25,26]. *SEPT4* can be a marker used for the diagnosis of human asthenozoospermia [27,28]. Moreover, *Sept4*-null mice are infertile because of defective morphology and immotile sperm [29,30]. Our previous studies have demonstrated that *SEPT12* is a testis-specific expressed gene. In both humans and mice, mutated *SEPT12* causes impaired sperm head morphology, bent tail, and DNA damage [18,31,32]. In addition, embryo development in mice fertilized with *Sept12*-deficient sperm through intracytoplasmic sperm injection ICSI arrests at the early morula stage [33].

### 1.3. SEPT14

*SEPT14* was originally identified as a novel testis-expressed protein [34]. Furthermore, *SEPT14* participates in cortical neuronal migration through cooperation with *SEPT4* [35]. In addition, two *SEPT14* genetic variants have been identified as reduced risk sites of Parkinson disease (PD) through the screening of 720 cases with PD and 740 controls [36]. In male reproduction, the expressional levels of *SEPT14* transcripts are required for human spermatogenesis through evaluating the testicular tissues of men with normal and defective, including hypospermatogenesis, maturation arrest, and sertoli cell–only syndrome [37]. Moreover, *SEPT14* is mainly localized in the front of the acrosome, neck, and tail [38]. The main objective of this study was to evaluate the potential genetic effects of *SEPT14* on male infertility through sequencing the *SEPT14* coding region.

## 2. Materials and Methods

### 2.1. Participants and Semen Analysis

This study was approved by the Ethics Committee of the Cathay General Hospital (IRB Approval No.: CGH-P102031). The participants were clinically diagnosed by semen analysis and further divided into two groups: controls with normozoospermia (*n* = 116; semen samples with a sperm concentration of ≥ 15 × 10^6^/mL, a progressive motility of >32%, and a normal morphology of >14%) and patients with abnormal semen parameter (*n* = 254). All participants signed a written informed consent form. Semen samples were obtained by masturbation after 3–5 days of sexual abstinence. After liquefying the semen at room temperature, routine semen analysis was performed according to the WHO laboratory manual for the examination and processing of human semen, 2010 (World Health Organization, 2010). Sperm morphology examinations were conducted in accordance with the Krueger strict criteria (normal morphology >14%).

### 2.2. Sperm Genomic DNA Extraction, Polymerase Chain Reaction, and Sequencing

A Wizard Genomic DNA Purification Kit (A1125, Promega, Madison, WI, USA) was used to extract genomic DNA from sperm. Sperm was washed in phosphate-buffered saline (PBS) and centrifuged at 300 *g* for 5 min twice. The sperm pellet was resuspended and incubated in lysis buffer, to which proteinase K (200 μg/mL) and dithiothreitol (150 mM) were added and left for 2–3 h at 56 °C. Genomic DNA was precipitated with isopropanol and washed in 70% ethanol twice. After centrifugation, DNA pellets were then air-dried and resuspended in nuclease-free ddH_2_O. The primers used in this study were designed for whole-coding regions and exon/intron boundaries of *SEPT14* (GenBank: NM_207366.2) and are listed in Table S1. After polymerase chain reaction (PCR), the nucleotide variants of the *SEPT14* coding region were analyzed through Sanger sequencing.

### 2.3. Transmission Electron Microscopy

Sperm was prepared for transmission electron microscopy (TEM) (JEM-1400; JEOL, Tokyo, Japan) as previously described [39]. Briefly, sperm was washed in PBS and centrifuged at 300 *g* for 5 min; this process was repeated twice. The sperm pellet was extremely gently mixed with 0.1% glutaraldehyde at 4 °C overnight. The fixed sperm pellet was rinsed with 0.1 M phosphate buffer (pH 7.2) and then treated with 1% osmium tetroxide at room temperature for 2 h. It was then rinsed again with phosphate buffer and progressively dehydrated through a series of ethanol treatments at increasing concentrations. The Spurr’s resin kit (cat-14300; Electron Microscopy Sciences, PA, USA) was used to embed the sperm pellet at room temperature overnight. Finally, 75-nm thin sections of the embedded samples were cut using an ultramicrotome (EM-UC7, Leica microsystems, Wetzlar, Germany) and were mounted on copper grids. Counter-staining was performed with uranyl acetate and lead citrate. An ultramicrograph was acquired using TEM (JEM-1400; JEOL, Tokyo, Japan) at 100 Kva.

### 2.4. Toluidine Blue Staining

Toluidine blue (TB) staining is a well-established method for examining chromatin integrity, and in this study, it was performed according to the protocol detailed in relevant studies [40]. TB is a basic metachromatic dye with a high binding ability to DNA phosphate residues. It is incorporated in the incomplete and poor packaging DNA structure of sperm and subsequently produces a blue to deep violet (purple) color. In this study, smears were prepared and fixed in freshly prepared 96% ethanol–acetone (1:1) at 4 °C from 30 min to 12 h before being air-dried. Subsequently, the smears were hydrolyzed in 0.1 N HCl at 4 °C for 5 min and rinsed three times in distilled water for 2 min. Finally, each smear was stained with 0.05% TB (89640, Sigma, St. Louis, MO, USA) in 50% McIlvaine’s citrate phosphate buffer (pH 3.5) for 5 min and then washed with distilled water. TB images were acquired through light microscopy, and for 150 sperm samples, various areas of each slide were examined under oil immersion with ×1000 magnification. Normal sperm with satisfactory chromatin levels was unstained or exhibited pale blue staining, and sperm with abnormal chromatin integrity was stained deep violet. The mean percentage of sperm with both head defects and diminished chromatin integrity in the control group was used as a reference to calculate the fold change in patients with *SEPT14* A123T or I333T mutations.

### 2.5. Comet Assay

Sperm DNA integrity was evaluated using the neutral comet assay [41]. After sperm was washed, the sperm suspension was adjusted to a concentration of 1 × 10^6^/mL in PBS and was mixed with 1% (w/v) low-melting-point agarose (Type VIIA; Sigma-Aldrich, MO, UK) at a ratio of 1:10 (v/v). The mixture was layered onto the surface of two microscope slides to form two microgels, and the slides were transferred to a refrigerator at 4 °C for 10 min. Microgels were submersed in a precooled lysis buffer (2.5 M NaCl, 100 mM EDTA, 10 mM Tris, 1% Triton X-100, and 40 mM dithiothreitol) for 1 h at room temperature and protected from light exposure. Following this initial lysis buffer submersion period, proteinase K (10 μg/mL) was added to the lysis solution, and the microgels underwent additional lysis at 37 °C for 2.5 h. Before electrophoresis, the slides were washed with ddH_2_O and placed in a horizontal electrophoresis unit, where they were equilibrated for 20 min with TBE buffer. After electrophoresis, the slides were washed twice with ddH_2_O and stained with SYBR staining solution. After staining, the slides were rinsed with ddH_2_O and then air-dried. A total of 100 sperm cells per semen sample were randomly selected from the two slides before analysis. Comet contents, including percentage of DNA in the tail (% DNA in the tail) and tail length, were measured using CometScore 2.0. The %DNA in tail measure indicates the proportion of total DNA that has migrated to the tail. Tail length refers to the distance from the fragmented DNA to the head.

### 2.6. Cloning, Transfection, and Immunofluorescence Staining

Full lengths of human *SEPTIN14* were reverse transcription-polymerase chain reaction (RT-PCR)-amplified from a human RNA panel and cloned into pFLAG-CMV2, as described previously [42]. The mutation constructs were prepared using QuikChange Site-directed Mutagenesis Kits (Stratagene, La Jolla, CA, USA). All constructs were confirmed by performing DNA sequencing. After HeLa cells were transfected with plasmids using Lipofectamine 2000 (Invitrogen, Carlsbad, CA, USA), the cells were subjected to immunofluorescence staining. The immunofluorescence staining process is described previously [32]. The cells were exposed to the anti-FLAG antibody (Sigma, F1804, St. Louis, MO, USA) at 4 °C overnight. After washing with PBST, the slides were incubated with secondary antibodies and stained with phalloidin and 4’,6-diamidino-2-phenylindole (DAPI). Subsequently, the slides were mounted on Dako fluorescence mounting medium.

### 2.7. Statistical Analysis

The quantification results are presented as mean ± standard error of the mean. The Student’s *t* test (two-tailed) was used to evaluate the significance between the two groups. Differences were considered statistically significant at *p* < 0.05.

## 3. Results

### 3.1. Two Novel Missense Mutations in the SEPT14 Gene

**Figure 1:**
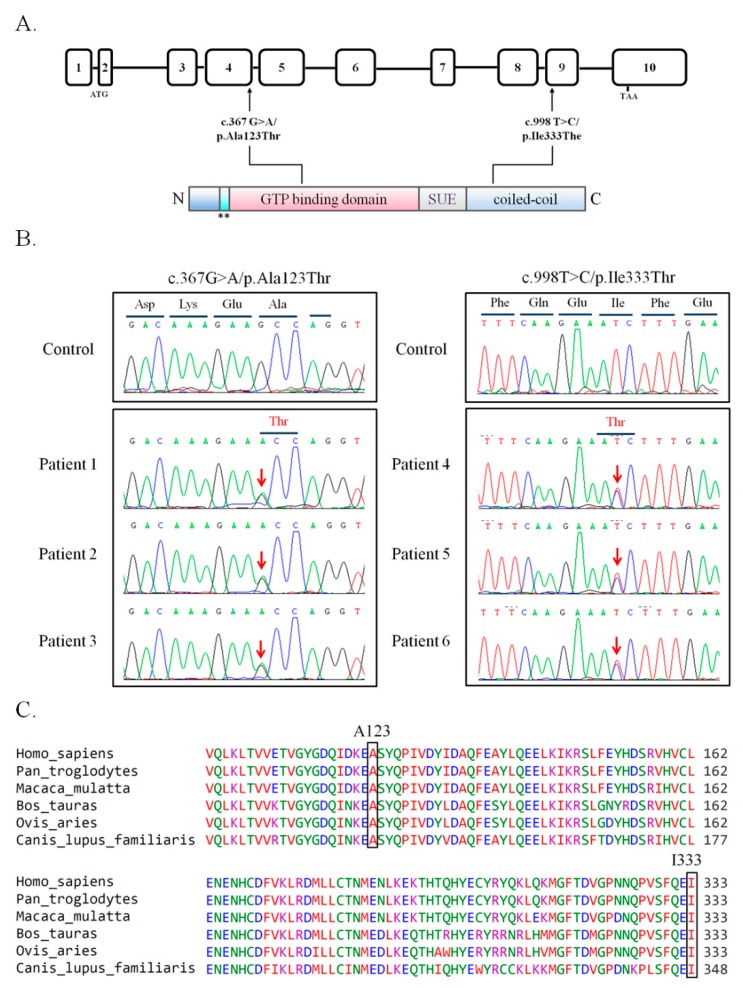
Identification of novel *SEPT14* missense mutations associated with male infertility. (**A**) Schematic of the *SEPT14* gene (upper panel) and protein (lower panel). Two missense mutations, A123T and I333T, were observed in patients with teratozoospermia. A123T was located in the GTP-binding domain, and I333T was located in the C-terminal coiled-coil domain. The 10 exons of the human *SEPT14* were numbered, and the start and stop codons were annotated. N = N-terminal; ** = phosphoinositide-binding polybasic region; SUE = septin unique element; C = C-terminal. (**B**) Sanger sequencing chromatograms of *SEPT14* mutations. Chromatograms display the sequence corresponding to WT and mutated alleles (upper and lower panels, respectively). These two mutations were noted in three patients and were heterozygous in each patient. Red arrows indicate nucleotide changes. (C) Evolutionary conservation of the p.A123 and p.I333 residues of the SEPT14 protein. Both A123 and I333 residues are highly conserved in various mammalian species. Alignment was performed with Clustal Omega (EMBL-EBI). Key residues, namely A123 and I333, are indicated by black squares. The numbers on the left indicate the amino acid number of the SEPT14 protein sequence.

To screen the possible genetic effects of *SEPT14* on male fertility, we collected semen samples from 254 male patients with abnormal semen parameter and 116 controls with normozoospermia. We designed the primers (Table S1) targeted to the whole-coding regions of *SEPT14* and performed Sanger sequencing to characterize unknown genetic variants of male infertility. Several variants located in exons and exon/intron boundaries were observed (Tables S2 and S3). Notably, two novel missense mutations (c.367G > A, *n* = 3; c.998T > C, *n* = 3) were noted in six cases with abnormal semen parameter (Table 1). The c.367G > A mutation was located in exon 4 and caused a substitution of alanine to threonine at position 123 (p.A123T) within the GTP-binding domain of *SEPT14* (Figure 1A,B). The other mutation, c.998T > C, was located in exon 9 and induced an amino acid change from isoleucine to threonine at position 333 (p.I333T) within the coiled-coil domain of *SEPT14* (Figure 1A,B). Both residues, A123 and I333, are evolutionarily highly conserved among vertebrates (Figure 1C). The clinical data of the six cases are summarized in Table 1. All cases carrying *SEPT14* mutations presented with teratozoospermia (91.5% ± 2.88% abnormal sperm), and morphological abnormalities were attributed to head defects (90% ± 4%). Therefore, we speculated that *SEPT14* mutations may disturb the morphology of sperm heads and cause male infertility.

### 3.2. Severely Malformed Heads and Diminished Chromatin Integrity in Sperm with SEPT14 Mutations

**Figure 2:**
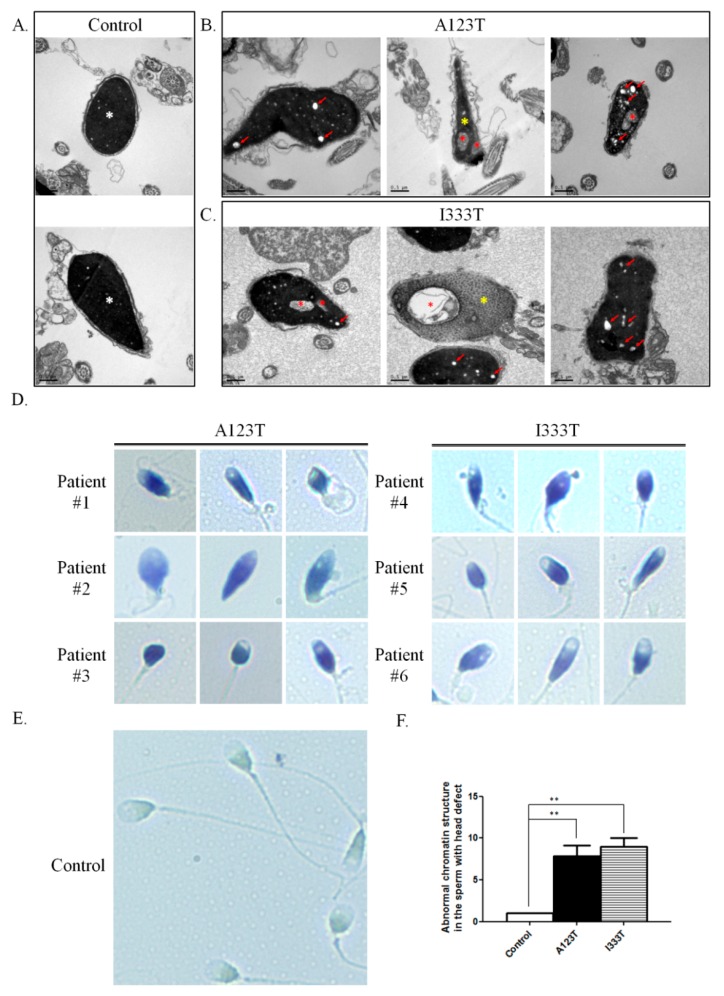
Sperm from patients with A123T or I333T exhibited severely malformed head shapes and aberrant chromatin integrity. (**A**–**C**) Electron microscopic morphology of the sperm heads from controls and cases with *SEPT14* mutations. Micrographs of various sections of the sperm heads from controls. A normal sperm head is oval with highly condensed chromatin (white asterisks) (**A**). Most of the sperm from cases with A123T (**B**) or I333T (**C**) exhibited head deformities and numerous nuclear vacuoles (red arrows or asterisks). Moreover, sperm with *SEPT14* mutations displayed chromatin packaging failure (yellow asterisk). (**D**–**F**) Representative photos of TB staining on sperm from each infertile patient with A123T (D, left panel) or I333T (D, right panel) mutations or controls (E). In sperm from patients with *SEPT14* mutations, most of the sperm with head defects was stained in deep violet because of the abnormal chromatin structure (**D**). In contrast to the abnormal sperm, sperm with satisfactory chromatin structure was unstained or stained pale blue in (**E**). (**F**) Quantification of sperm with abnormal chromatin integrity. All quantifications were performed on the control (*n* = 6), A123T (*n* = 3), and I333T (*n* = 3) groups. (** *p* < 0.01, analyzed using Student’s *t* test).

To clarify the detailed morphological effects of *SEPT14* mutations on the structure of sperm heads, TEM was used. Compared with controls, sperm from patients carrying A123T or I333T showed prominently malformed heads and contained numerous vacuoles (Figure 2A–C). Furthermore, some sperm samples exhibited abnormal chromatin packaging (Figure 2B, yellow asterisks). Since high chromatin quality is critical for fertilization and achieving successful pregnancy outcomes, we also examined the chromatin integrity of sperm from the patients carrying *SEPT14* mutations through TB staining. Based on TB staining, the ratio of the abnormal chromatin structure (deep violet) in sperm with head defects of *SEPT14*-mutated cases increased significantly compared with controls (light blue) (Figure 2D–F). According to these results, we suggest that *SEPT14* mutations impair sperm nuclear morphology and chromatin integrity.

### 3.3. DNA Integrity in Sperm with SEPT14 Mutations

**Figure 3:**
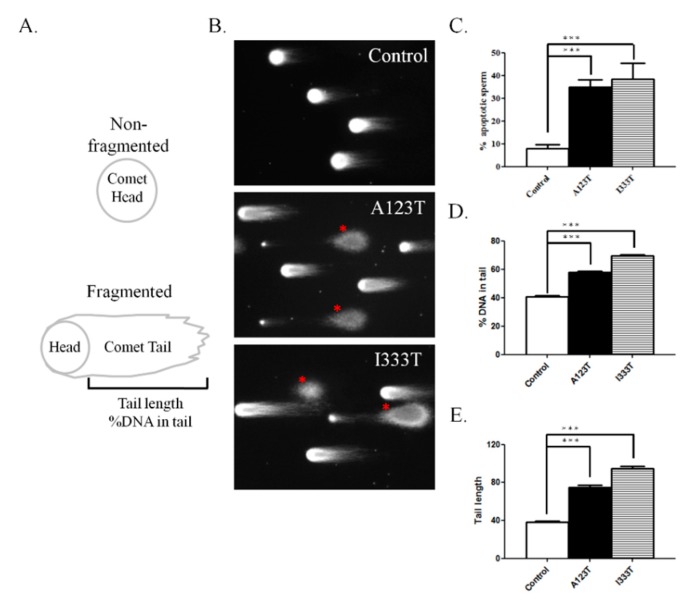
Infertile patients with *SEPT14* mutations have significantly higher DNA fragmentation than controls. (**A**) Illustration of the comet content. DNA fragments migrated out of the heads and resemble a “comet tail” under electrophoresis. The intensity and length of the comet tail reflect the degree of DNA damage. (**B**) Representative images of DNA damage in sperm obtained from controls and patients with A123T or I333T. (**C**) Quantification of % apoptotic sperm number. (**D** and E) Quantification of %DNA in the tail and tail length. Evident DNA damage was observed in sperm from infertile patients with *SEPT14* mutations. All quantifications were performed on the control (*n* = 6), A123T (*n* = 3), and I333T (*n* = 3) groups. * Significant difference compared with controls (***, *p* < 0.001, analyzed using Student’s *t* test).

Although a high percentage of sperm with abnormal head morphology and the loss of chromatin packing was found in cases with *SEPT14* mutations, the level of DNA damage/fragmentation of sperm remains unknown. To address this concern, the comet assay was used. Two indices of comet assay, namely the percentage of fragmented DNA in the tail and the tail length of comet, were evaluated in the sperm samples through the comet assay (Figure 3A). Figure 3B,C reveal that sperm carrying *SEPT14* A123T or I333T is full of DNA fragments, with a high percentage of fragmented DNA in the tail and a longer tail length, compared with controls. Based on these results, we suggest *SEPT14* mutations damage DNA integrity in sperm.

### 3.4. SEPT14 Mutations Damage the Forming Ability of the Filamentous Structure

**Figure 4:**
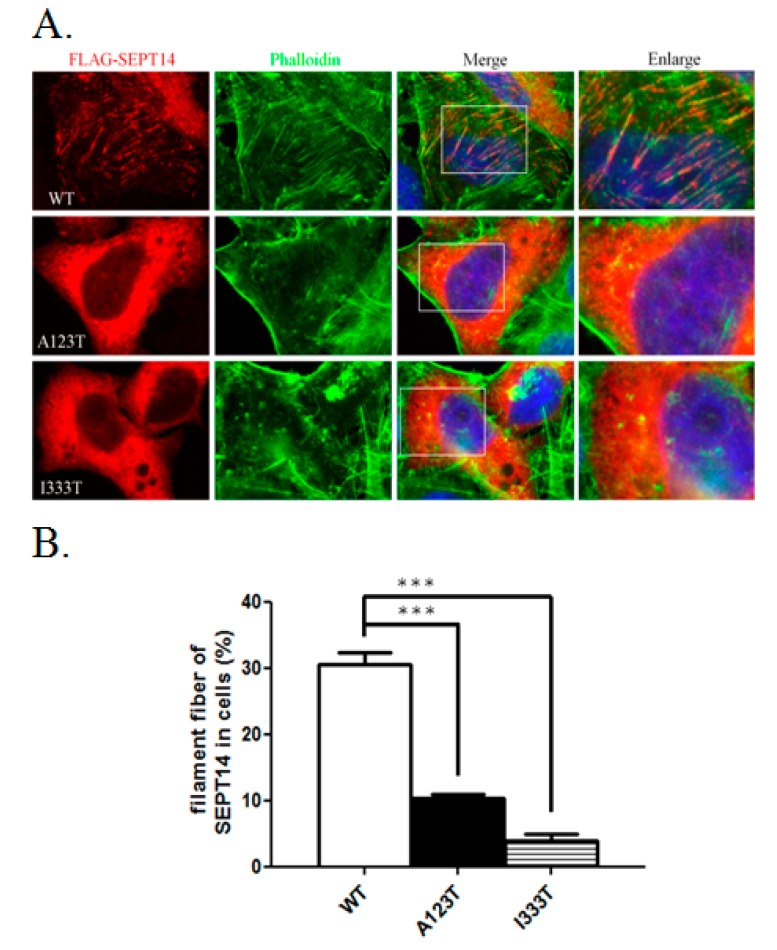
Filament forming ability of WT-*SEPT14* and mutant SEPT14. (**A**) Immunofluorescence staining of the cells transfected with plasmids encoding WT FLAG-*SEPT14* (WT panel), FLAG-*SEPT14*^A123T^ (A123T panel), and FLAG-*SEPT14*^I333T^ (I333T panel). From left to right: Staining with anti-FLAG antibody (*SEPT14*), staining with phalloidin (phalloidin), merging with the figures of staining with anti-FLAG antibody, phalloidin, and DAPI (merge), and enlarged figure (enlarge). (**B**) The quantification bar chart was based on the observation of more than 100 cells for each experiment in Figure 4A. Filament fibers indicate the percentages of cells with *SEPT14* filaments. (*p* < 0.01, Student’s *t* test comparing filament fibers to others). Data are represented as means ± standard error of three tests.

To determine whether *SEPT14* mutations affect its cellular function e.g., filamentous formation, wild-type (WT) and mutant *SEPT14* constructs were transfected into the cells, followed by immunofluorescence staining (Figure 4). Figure 4A shows that FLAG-*SEPT14* (WT) forms the filamentous structure (red) and is colocalized with F-actin (green) in the cell, as revealed by staining with phalloidin. Furthermore, compared with FLAG-*SEPT14* (WT), only few of the filament structures observed in the cell were transfected with the mutated SEPT14 (FLAG-*SEPT14*^A123T^ and FLAG-*SEPT14*^I333T^) constructs. Through quantitative estimation from three independent assays, Figure 4B reveals that the percentage of formed filaments of the mutated *SEPT14* constructs decreased significantly compared with FLAG-*SEPT14* (WT). This result demonstrates that mutated *SEPT14* damages the forming ability of the filamentous structure.

## 4. Discussion

*SEPT14* is the last identified gene in the *SEPTIN* family and is abundantly expressed in testes and neurons. In this study, we characterized the genetic effects of *SEPT14* on cases with abnormal sperm parameters by sequencing the whole-coding regions of *SEPT14.* We identified two mutations, A123T and I333T, in teratozoospermia cases. Moreover, sperm from cases carrying *SEPT14* mutations not only displayed severe structural defects of the sperm head but also presented with high levels of DNA damage. In addition, these mutations affected the polymerization ability of *SEPT14* in vitro. This is the first study to reveal the genetic alternations and spermatogenetic functions of *SEPT14* in maintaining human sperm head morphology and nuclear DNA integration.

### 4.1. SEPTs and Sperm DNA Damage

We previously discovered two heterozygous missense mutations of *SEPT12*, in which threonine was replaced with methionine at amino acid 89 (T89M; 1/160) and aspartate was replaced with asparagine at amino acid 197 (D197N; 1/160), from screening 160 male patients with infertility [31]. Both mutations were located within the GTPase domain, a critical domain for binding GTP for *SEPT* polymerization. However, sperm from patients with *SEPT12*D197N presented a defective annulus between the midpiece and the principal piece of the sperm tail, with a bent tail. Moreover, we discovered a *SEPT12* variant (c.474 G > A; 15/160), which affects RNA splicing and creates a truncated C-terminal of *SEPT12*. It inhibits *SEPT12* polymerization considerably. Approximately 88%–99% of sperm from infertile men carrying the c.474 G > A SNP display severe sperm head defects, with DNA damage and nuclear defects [18]. Due to the dissimilar phenotypes between sperm with mutations and c.474 G > A variants, we speculate that *SEPT12* has multiple functions during sperm head and sperm tail annulus formation. In this study, two missense mutations (A123T, 3/254; I333T, 3/254) of *SEPT14* were observed in treatozoospermia cases with severe sperm head defects (Table 1 and Figure 1). Sperm from these patients exhibited a disrupted ultrastructure of sperm heads and DNA damage (Figure 2 and Figure 3). Moreover, the mutations of *SEPT14* decreased its polymerization ability (Figure 4). The present study supports that mutated *SEPT14* affects sperm head formation and causes DNA damage through the loss of polymerization ability.

### 4.2. Sperm DNA Damage and ART

Sperm DNA damage may be attributable to factors such as cigarette smoking, chemotherapy or radiation therapy, environmental toxins, genital tract inflammation, reactive oxygen species, testicular hyperthermia, hormonal factors, and gene mutations [12,43,44]. Many studies indicated high level of sperm DNA damage is related to a high miscarriage rate, decreased pregnancy rate [45,46,47,48,49,50,51]. In our previous study, sperm with the defective *Sept12* allele exhibited abnormal sperm heads and increased DNA damage [32,33]. Mouse embryo development through injected oocyte with *Sept12*-deficient sperm arrests at the early morula stage [33]. In the present study, we also demonstrated that sperm with mutated *SEPT14* exhibited abnormal sperm heads and increased DNA damage. We suggest that mutated *SEPT14* is one of the causes of sperm DNA damage in infertile men and may be an indicator for the successful rates of assisted reproductive technology (ART).

## 5. Conclusions

This study is the first to link the genetic changes of *SEPT14* to sperm DNA damage and male infertility.

## Figures and Tables

**Table 1:** Clinical characteristics of infertile patients with c.367G > A/p.Ala123Thr and c.998T > C/p.Ile333Thr *SEPTIN14* mutations.

	Nucleotide Sequence/Amino Acid Variation					
	c.367G>A/p.Ala123Thr			c.998T>C/p.Ile333Thr		
Ratio	Patients: 3/254; Controls: 0/116			Patients: 3/254; Controls: 0/116		
Exon		4			9	
Case No.	Patient 1	Patient 2	Patient 3	Patient 4	Patient 5	Patient 6
Clinical feature	Oligoteratozoospermia	Teratozoospermia	Teratozoospermia	Teratozoospermia	Teratozoospermia	Teratozoospermia
Abnormal morphology (%) (Krueger criteria)	90	88	95	91	90	95
Head defect (%)	91	83	91	88	93	94
Neck defect (%)	1	9	3	9	4	3
Tail defect (%)	9	11	10	11	5	18
Immature (%)	11	13	1	3	3	5
Sperm concentration (10^6^/ml) (>15 × 10^6^/mL)	13	145	70	28	68	20
Progressive motility (%) (>32%)	49	55	37	48	54	45
Non-progressive motility (%)	15	11	16	17	14	30
Karyotype	46, XY	46, XY	46, XY	46, XY	46, XY	46, XY
Age (years)	31	39	35	32	35	38
Cigarette smoking, alcohol consumption, genital disease	No	No	No	No	No	No

## Supplementary Materials

The following are available online at https://www.mdpi.com/2077-0383/8/9/1297/s1, Table S1: Sequences of primers used in this study for *SEPTIN14* novel variants, Table S2: Variants identified in infertile men in the coding region of *SEPTIN14*, Table S3: Variants identified in infertile men in the noncoding regions of the *SEPTIN14* gene.
