# Supplementary material for: SEPT14 Mutations and Teratozoospermia: Genetic Effects on Sperm Head Morphology and DNA Integrity
Source: J Clin Med. 2019 Aug 23;8(9):1297. doi: 10.3390/jcm8091297 (PMC6780492; doi:10.3390/jcm8091297)
Supplement: Supplementary file 1 [file jcm-08-01297-s001.pdf]

Table S1. Sequences of primers used in this study for *SEPTIN14* novel variants.

Exon		Primer	Product size (bp)	Annealing temperature (℃)
2	Forward	AAACAGATGAATAGTGTGAAC	423	56
	Reverse	CTAACAAGGTCATCGGAC		
3	Forward	GCATCTTGTCTGAGGTATG	431	58
	Reverse	CAAAGTGAGAACCTGTCT		
4	Forward	GACCAACTACTCCACCTTTCC	789	59
	Reverse	GCCTGGGTGATGGAATGAAA		
5	Forward	GCACCCAGCTTTCCTCTATG	763	58
	Reverse	GCAAGACCCTGCCTCAAATA		
6	Forward	CAGGAGAGTGGCGTGAAC	587	56
	Reverse	GGTAACACCTTCAGAGTCATTT		
7	Forward	TGCAGGGTAGATCCCTGTCTA	743	58
	Reverse	GCAGCGTTCAGCAAACTTCT		
8	Forward	TTGTTCAGTTGTGTGTTATT	403	56
	Reverse	TTATGATGGCTCAAGATGG		
9	Forward	GTCTTACTCTGTCTCCCA	413	58
	Reverse	AAGGCTATATTCAAGTCTACA		
10	Forward	TATCTCAGTGTTTACTCCTG	407	54
	Reverse	ATGCTACAAAGACAATCTCA		

Table S2. Variants identified in infertile men in the coding region of *SEPTIN14*.

variants	Genotype	Genotype frequency		rs No./ Functional Consequence
		Case (n=254)	Control (n=116)	
c.116G>A	GG	251 (98.8 %)	112 (96.6 %)	
p.Pro39His	GA	3 (1.2 %)	4 (3.4 %)	
	AA	0 (0.0 %)	0 (0.0 %)	
c.367G>A	GG	251 (98.8 %)	116 (100.0 %)	
p.Ala123Thr	GA	3 (1.2 %)	0 (0.0 %)	
	AA	0 (0.0 %)	0 (0.0 %)	
c.459C>T	CC	254 (100.0 %)	114 (98.3 %)	rs202157028
p.Tyr153=	CT	0 (0.0 %)	2 (1.7 %)	synonymous codon
	TT	0 (0.0 %)	0 (0.0 %)	
c.543G>A	GG	253 (99.6 %)	116 (100.0 %)	
p.Lys181=	GA	1 (0.4 %)	0 (0.0 %)	
	AA	0 (0.0 %)	0 (0.0 %)	
c.617C>T	CC	254 (100.0 %)	115 (99.1 %)	rs761205837
p.Thr206Met	CT	0 (0.0 %)	1 (0.9 %)	missense
	TT	0 (0.0 %)	0 (0.0 %)	
c.998T>C	TT	251 (98.8 %)	116 (100.0 %)	rs185254020
p.Ile333Thr	TC	3 (1.2 %)	0 (0.0 %)	missense
	CC	0 (0.0 %)	0 (0.0 %)	

Table S3. Variants identified in infertile men in the noncoding regions of the *SEPTIN14* gene.

variants	Genotype frequency			rs No./ Functional Consequence
	Genotype	Case (n=254)	Control (n=116)	
c.-15-42T>C	TT	249 (98.0 %)	113 (97.4 %)	rs1212548424
	TC	5 (2.0 %)	3 (2.6 %)	intron variant
	CC	0 (0.0 %)	0 (0.0 %)	
c.54+97A>T	AA	75 (29.5 %)	39 (33.6 %)	rs10499759
	AT	127 (50.0 %)	58 (50.0 %)	intron variant
	TT	52 (20.5 %)	19 (16.4 %)	
c.176-41C>A	CC	253 (99.6 %)	116 (100.0 %)	
	CA	1 (0.4 %)	0 (0.0 %)	
	AA	0 (0.0 %)	0 (0.0 %)	
c.559-120C>T	CC	249 (98.0 %)	115 (99.1 %)	rs185656898
	CT	5 (2.0 %)	1 (0.9 %)	intron variant
	TT	0 (0.0 %)	0 (0.0 %)	
c.721-291C>T	CC	192 (75.6 %)	89 (76.7 %)	rs138074720
	CT	56 (22.0 %)	25 (21.6 %)	intron variant
	TT	6 (2.4 %)	2 (1.7 %)	
c.721-222G>A	GG	104 (40.9 %)	53 (45.7 %)	rs11767417
	GA	112 (44.1 %)	46 (39.7 %)	intron variant
	AA	38 (15.0 %)	17 (14.7 %)	
c.817+26G>T	GG	192 (75.6 %)	84 (72.4 %)	rs73701152
	GT	52 (20.5 %)	26 (22.4 %)	intron variant
	TT	10 (3.9 %)	6 (5.2 %)	
c.721-314G>A	GG	254 (100.0 %)	115 (99.1 %)	rs1363256558
	GA	0 (0.0 %)	1 (0.9 %)	intron variant
	AA	0 (0.0 %)	0 (0.0 %)	
c.721-234G>A	GG	253 (99.6 %)	116 (100.0 %)	rs373047198
	GA	1 (0.4 %)	0 (0.0 %)	intron variant
	AA	0 (0.0 %)	0 (0.0 %)	
c.986+57A>C	AA	247 (97.2 %)	112 (96.6 %)	rs78233970
	AC	7 (2.8 %)	4 (3.4 %)	intron variant
	CC	0 (0.0 %)	0 (0.0 %)	
c.987-32T>A	TT	253 (99.6 %)	116 (100.0 %)	
	TA	1 (0.4 %)	0 (0.0 %)	
	AA	0 (0.0 %)	0 (0.0 %)	
